# Comprehensive Genetic Testing of *CYP21A2*: A Retrospective Analysis in Patients with Suspected Congenital Adrenal Hyperplasia

**DOI:** 10.3390/jcm10061183

**Published:** 2021-03-12

**Authors:** Madalina Nicoleta Nan, Rosa Roig, Susana Martínez, Jose Rives, Eulàlia Urgell, Juan José Espinós, Mireia Tirado, Gemma Carreras, Anna Aulinas, Susan M. Webb, Rosa Corcoy, Francisco Blanco-Vaca, Mireia Tondo

**Affiliations:** 1 Servei de Bioquímica, Hospital de la Santa Creu i Sant Pau—IIB Sant Pau, 08041 Barcelona, Spain; mnan@santpau.cat (M.N.N.); rroig@santpau.cat (R.R.); smartinezf@santpau.cat (S.M.); jrives@santpau.cat (J.R.); eurgell@santpau.cat (E.U.); fblancova@santpau.cat (F.B.-V.); 2 Servei de Ginecologia, Hospital de la Santa Creu i Sant Pau—IIB Sant Pau, 08041 Barcelona, Spain; jespinos@santpau.cat; 3 Servei de Pediatria, Hospital de la Santa Creu i Sant Pau—IIB Sant Pau, 08041 Barcelona, Spain; mtirado@santpau.cat (M.T.); gcarreras@santpau.ca (G.C.); 4 Servei d’Endocrinologia, Hospital de la Santa Creu i Sant Pau—IIB Sant Pau, 08041 Barcelona, Spain; aaulinas@santpau.cat (A.A.); swebb@santpau.cat (S.M.W.); rcorcoy@santpau.cat (R.C.); 5 Facultat de Medicina, Universitat de Vic—Universitat Central de Catalunya, 08500 Vic, Spain; 6 CIBER de Enfermedades Raras (CIBERER), ISCIII, 28029 Madrid, Spain; 7 Departament de Medicina, Universitat Autònoma de Barcelona, 08193 Barcelona, Spain; 8 CIBER de Bioingeniería, Biomateriales y Nanomedicina (CIBERBBN), ISCIII, 28029 Madrid, Spain; 9 CIBER de Diabetes y Enfermedades Metabólicas Asociadas (CIBERDEM), ISCIII, 28029 Madrid, Spain; 10 Departament de Bioquímica i Biologia Molecular, Universitat Autònoma de Barcelona, 08193 Barcelona, Spain

**Keywords:** congenital adrenal hyperplasia, *CYP21A2* gene, genotype analysis, 17-OH progesterone, genotype-phenotype correlation

## Abstract

The most common form of congenital adrenal hyperplasia (CAH) results from a deficiency of the 21-hydroxylase enzyme (21-OHD), presenting with a broad spectrum of clinical phenotypes according to the *CYP21A2* gene mutations. Of the 59 patients with suspected CAH, 62.7% presented a positive genetic result. Of them, 78.4% and 18.9% presented with non-classical and classical forms, respectively. An overall phenotype-genotype correlation of 88.9% was observed. Biochemically, 17-hydroxiprogesterone concentrations were significantly higher in genetically confirmed patients. Genetically, 36 patients presented with previously reported pathogenic variants, and one presented a new variant in homozygosis. Among the 74 alleles tested, point mutations were found in 89.2% and large rearrangements were found in the rest. The most prevalent pathogenic variant was p.(Val282Leu). The inclusion of relatives revealed one further case. Interestingly, 87.5% of relatives were carriers of a pathogenic variant, including two siblings initially classified as genetically positive. In addition, the study of male partners with gestational desire identified several carriers of mild mutations. Studying the allelic distribution of the variants also allowed for reclassifying one patient. In conclusion, a genetic approach including Sanger sequencing, multiplex ligation-dependent probe amplification (MLPA) analysis, and allelic distribution of the pathogenic variants represents a beneficial tool for better classifying patients with 21-OHD.

## 1. Introduction

Congenital adrenal hyperplasia (CAH) comprises a group of autosomal recessive disorders whose most common form results from a deficiency of the 21-hydroxylase enzyme (21-OHD; #OMIM 201910), which accounts for about 90–95% of cases [1]. 21-OHD presents a broad spectrum of clinical phenotypes according to the *CYP21A2* gene mutations on the 21-hydroxylase enzyme (21-OH). Classification goes from severe or classical (CL) forms that manifest earlier in life, or even prenatally, to mild late-onset non-classical (NC) forms. CL forms include a salt-wasting (SW) phenotype, with an impossibility to synthesize aldosterone, and a simple-virilizing (SV) phenotype, with signs of androgen excess due to the mild-to-moderate overproduction of sex hormones, but with normal aldosterone synthesis [2]. Key features of the CL form in newborns are ambiguous genitalia in females, neonatal salt loss, failure to thrive, and potentially fatal hypovolemia and shock. Later in life, it is characterized by rapid postnatal growth, sexual precocity, different signs of hyperandrogenism, and reduced fertility. The NC form presents with premature pubarche, hirsutism, acne, menstrual abnormalities, unfulfilled pregnancy, or no clinical symptoms (cryptic CAH) [3].

The prevalence of 21-OHD, as well as its mutation pattern, varies among different ethnic populations. Several studies estimate that worldwide, incidence is 1:10,000 to 1:15,000 births for the CL form and 1:100 to 1:1000 births for the NC form [4,5,6]. A study carried out in a Spanish population found a heterozygote frequency of 1:6.5, with an estimated incidence of 1:225 for the NC form [7].

The *CYP21A2* gene is located in the HLA class III region in chromosome 6p21.3, together with a highly homologous inactive pseudogene (*CYP21A1P*) [8]. In conjunction with neighboring genes and their truncated pseudogenes, they constitute a highly variable genetic unit termed the RCCX module. Most chromosomes carry two modules; however, chromosomes carrying one, three, or even four modules have been described [7]. The high genetic variability at the *CYP21A2* locus makes the characterization of 21-OH alleles difficult, complicating disease-carrier detection and genetic counseling. To date, more than 300 pathogenic variants in the *CYP21A2* gene have been described (http://www.hgmd.cf.ac.uk, last accessed in 15 January 2021), 95% of them (including point mutations, large rearrangements, and gene deletions) due to recombination events between the gene and pseudogene.

Regarding the Spanish population, multiple works exploring the *CYP21A2* locus and its correlation with genotypes exist [7,9,10,11,12]. Our group also reported that a genetic analysis of *CYP21A2* did not confirm NC forms in more than a third of the women with this diagnosis [13]. In the present work, we explore not only the presence of small mutations and large rearrangements, but also the allelic distribution of the mutated alleles. 

Altogether, we present a comprehensive molecular genetic analysis of our cohort over the last 10 years following the implementation of genetic diagnosis, with the aim of generating a beneficial tool for the accurate diagnosis and genetic counseling of patients with 21-OHD.

## 2. Materials and Methods

### 2.1. Study Participants

This was a single-center descriptive study of adults and children with suspected CAH recruited at Hospital de la Santa Creu i Sant Pau in Barcelona, Spain, over a 10-year period (2010–2020). Patients were referred to our Metabolism Unit for genetic analysis of the *CYP21A2* gene based on biochemical and/or clinical suspicion of CAH, according to the requesting practitioners from the Endocrinology, Gynecology, and Pediatrics Units.

Compatible clinical features of CAH in our population included features of renal salt-wasting and adrenal crisis (hypovolemia, hyponatremia, hyperkalemia, fever, tachycardia, low arterial tension, loss of appetite, vomiting, nausea, intense prostration, loss of consciousness, and shock), ambiguous genitalia in females, premature pubarche, hirsutism, acne, menstrual abnormalities, and infertility. In all cases, genetic studies were performed to confirm the diagnosis. On some occasions, blood relatives and partners with gestational desire were also submitted for study. Overall, 59 patients with suspected CAH, 23 blood-related relatives, and 10 partners were submitted for genetic testing.

### 2.2. Ethical Aspects

The study was performed following the standards for medical research in humans recommended by the Declaration of Helsinki. All participants or their legally authorized representatives gave written informed consent.

### 2.3. Biochemical Parameters

Whole blood samples were collected by venipuncture in Vacutainer^TM^ tubes after an overnight fast, and were fractionated by centrifugation at 1300 g for 15 min at room temperature to obtain serum. Serum was aliquoted into 1.5 mL tubes, and 17-OHP was measured according to a standard commercially available ELISA kit (DRG Diagnostics EIA-1292) adapted to a DYNEX DSX 4-plate ELISA processing system with an upper limit of quantification at 60.6 nmol/L. No dilutions were performed for samples above this limit.

The interpretation of the patients’ hormonal work-up was performed according to Speiser et al.: basal or stimulated 17-OHP ≥ 30 nmol/L was considered indicative of CAH, while basal 17-OHP ≥ 6 and < 30 nmol/L was considered abnormal, requiring further testing after stimulation with cosyntropin (synthetic adrenocorticotropic hormone (ACTH)) [14]. Cut-off values at younger ages require cautious interpretation, as 17-OHP at birth is usually higher [15]. In our cohort, measurement of 17-OHP was performed at 0 min (basal) and, when required, after cosyntropin administration 60 min later; the 17-OHP of women at reproductive age was quantified in the early follicular phase of the menstrual cycle.

### 2.4. Genetic Screening

Genomic DNA was isolated from whole blood (usually EDTA or citrate blood) using the QIAamp DNA blood minikit (Qiagen, Hilden, Germany). To avoid the coamplification of the pseudogene, special primers containing characteristic single nucleotide polymorphisms (SNP) of the *CYP21A2* gene were designed. Moreover, amplification of the *CYP21A2* gene by PCR was performed in only two fragments in order to avoid unspecific annealing. Fragment 1 covered 1019 bp from the promoter to exon 3, and fragment 2 covered 2189 bp from intron 2 to the 3′UTR. The designed primer sequences can be found in Table S1.

The resulting products were purified using GFX PCR DNA and a Gel Band Purification Kit (GE Healthcare, Buckinghamshire, U.K.), and were sequenced using a Big Dye Terminator cycle sequencing kit v.3.1 (Applied Biosystems, Foster, CA, USA) on an ABI3130XL automated analyzer (Applied Biosystems). The resulting electropherograms were analyzed with the Staden package program [16] according to the transcript reference NM_000500.9. Screening for large rearrangements (deletions, duplications, and conversions) was performed by multiplex ligation-dependent probe amplification (MLPA) using the P050-C1 CAH kit (MRC-Holland^®^ Amsterdam, The Netherlands).

To assess the allelic distribution of the pathogenic variants, five primers containing one of the variants detected in the patient were designed (Figure S1). When analyzing the results, if both the target and the pathogenic variants appeared in the amplified sequence, the distribution was in cis (same allele). On the contrary, if only the target variant appeared in the amplified sequence, the distribution was in trans (different alleles).

### 2.5. Characterization of Variants and Bioinformatics Analysis

The nomenclature of the allelic variants follows the recommendations of the Human Molecular Genome Variation Society (http://www.hgvs.org, last accessed in 15 January 2021). In order to characterize the variants, they were checked against the Human Gene Mutation Database (HGMD; www.hgmd.cf.ac.uk, last accessed in 15 January 2021), ClinVar (www.ncbi.nlm.nih.gov/clinvar, last accessed in 15 January 2021), and EMQN best-practice guidelines for molecular genetic testing and reporting of 21-OHD [17]. For the first-time reported variants, bioinformatics analysis was used. The impact of point mutations on the structure and function of the protein was assessed with the following software: SIFT (sift.bii.a-star.edu.sg, last accessed in 15 November 2020), PolyPhen2 (genetics.bwh.harvard.edu/pph2/index.shtml, last accessed in 15 November 2020), Mutation Taster (www.mutationtaster.org, last accessed in 15 November 2020), and Align-GVGD (www.agvgd.hci.utah.edu/, last accessed in 15 November 2020).

### 2.6. Statistical Analysis

Descriptive statistics were used to summarize the characteristics of the study population. Data normality was analyzed using the Kolmogorov-Smirnov test. Continuous variables were compared between study groups. A Student’s t-test and a Mann-Whitney test were used for parameters following a normal and a non-normal distribution, respectively. Data are presented as mean ± standard deviation (SD) for continuous variables and median (interquartile range (IQR)) for non-continuous variables. A *p*-value < 0.05 was considered statistically significant. The statistical software GraphPad Prism (GraphPad Software Inc., La Jolla, CA, USA) was used for statistical analyses.

## 3. Results

### 3.1. Study Cohort Characteristics

The study included 59 Spanish patients of Caucasian origin, except for two patients from the Dominican Republic and Argentina. Our cohort had 7 males and 52 females aged 2 days to 37 years (16.8 ± 11.7 years), of which 37 individuals (62.7%) presented a positive genetic result for CAH due to alterations in the *CYP21A2* gene. Of them, 5 (13.5%) were males and 32 (86.5%) were females, aged 3 days to 37 years (17.5 ± 12.9 years). The mean age at diagnosis for the SW, SV, and NC phenotypes and patients with a negative genetic diagnosis was 0.043 (0.035), 16.5 (21.9), 21.3 (10.9), and 15.7 (9.1) years, respectively. Of the patients with a confirmatory genetic result, 29 (78.4%) were diagnosed with NC CAH and 7 (18.9%) with CL CAH, including 5 patients with a SW phenotype and 2 with a SV one. One further patient presented with a non-definite phenotype in-between SV and NC forms. Clinically and at the time of diagnosis, signs of adrenal crisis were observed in only SW forms. Ambiguous genitalia were observed in female CL forms, as well as in some patients in which genetic diagnoses could not be achieved. Signs of early puberty, hirsutism, acne, menstrual abnormalities, and infertility were observed in both NC forms and negative genetic diagnosis. Regarding the follow-up and treatment of the 37 genetically confirmed CAH patients, 22 of them were followed-up in the hospital, 14 were not (most of them are being followed-up in primary healthcare facilities), and one further patient was recently diagnosed and no follow-up data were available. Further information on treatment and follow-up can be found in Table S2.

Regarding 17-OHP, the median basal 17-OHP was statistically higher in genetically confirmed CAH patients when compared to patients without a genetic diagnosis (42.08 (19.79–60.60) nmol/L, *n* = 35, versus 6.21 (3.05–8.75) nmol/L, *n* = 21; *p* < 0.0001). No statistical differences were observed between CAH groups regarding 17-OHP concentration. After stimulation with synthetic ACTH, the 17-OHP concentrations were statistically higher for patients diagnosed with a NC form than for genetically negative patients (60.60 (60.60–60.60) nmol/L, *n* = 12 versus 16.3 (7.7–19.8) nmol/L, *n* = 15; *p* < 0.0001). 

Interestingly, among the genetically negative patients, those carrying a mutated allele tended to present higher concentrations of 17-OHP after stimulation with ACTH than those that did not, although no statistical differences were found (17.88 ± 8.06 nmol/L, *n* = 8 versus 10.88 ± 5.63 nmol/L, *n* = 7; *p* = 0.077). The available clinical, biochemical, and mutation status of the cohort is presented in Table 1.

### 3.2. Genetic Results

**Figure 1:**
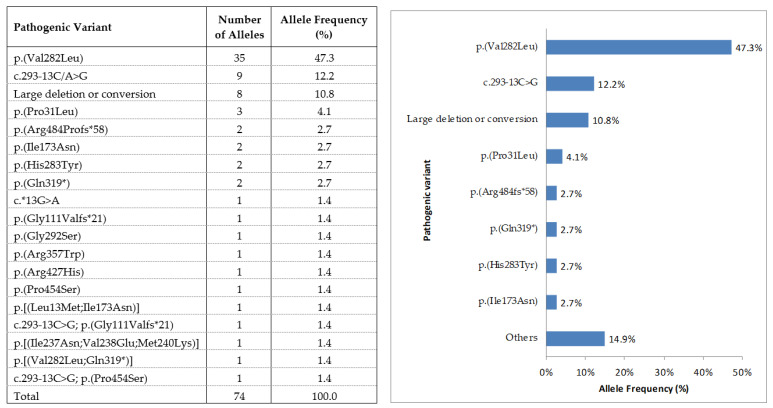
Mutation frequency of the 74 affected alleles from 37 unrelated patients with 21-hydroxylase enzyme (21-OH).

Among the 37 genetically confirmed patients, 36 presented with previously reported pathogenic variants and 1 presented a new variant in homozygosis. All of the consulted in silico software predicted the variant as pathogenic, and therefore a positive genetic diagnosis was performed. Of the 74 affected alleles, point mutations were found in 66 (89.2%), and large rearrangements were found in the remaining 8 (10.8%). The most prevalent pathogenic variant in our cohort was p.(Val282Leu), found in 35 alleles (47.3%), followed by c.293-13C>G found in 9 alleles (12.2%), and large deletion or conversion carried by 8 alleles (10.8%; Figure 1).

Regarding the genotype, 21 patients (56.8%) were compound heterozygotes, 10 (27.0%) were homozygous, and 6 (16.2%) were hemizygotes. The p.[(Val282Leu)];[(Val282Leu)] genotype was observed in nine patients (24.3%), followed by p.[(Val282Leu)];c.[293-13C>G] and p.[(Val282Leu)];c.[(-1)-(1118+1_119-1)del] observed in three patients each (8.1%). Patients’ pathogenic variants were classified according to their predicted enzymatic activity, following previously reported works [18,19,20].

In this regard, one patient carried variants reported to have null enzymatic activity and expected SW phenotype, five patients carried variants with < 2% enzymatic activity and expected SW phenotype, two patients carried variants with around 2% of enzymatic activity and expected SV phenotype, and 28 patients carried variants with 20–60% of enzymatic activity and expected NC phenotype. One further patient presented a new variant in homozygosis and therefore an unpredictable phenotype. Overall, of the 36 patients with an existing prediction of severity data, 32 patients (88.9%) presented agreement between the observed and expected phenotypes. Of the remaining four patients (11.1%) with a discordant phenotype, the patient carrying the c.[(-1)_(202+1_203-1)del];[(-1)_(202+1_203-1)del;(651+1_652-1)_(313+1_314-1)del] genotype was a girl diagnosed at 11 months of age with SV CAH. She never presented with signs of salt loss. She underwent genitoplasty surgery, including vaginoplasty and labia minora surgery at age 14, and vulvar repair and residual corpus cavernosum amputation at age 15. The patient carrying the c.[293-13C>G];p.[(Ile237Asn;Val238Glu;Met240Lys)] pathogenic variants was a man born in 1940 and was diagnosed at 33 years old with SV CAH. No signs of salt loss were ever observed. He was later identified with a 46XX karyotype and diagnosed with 46,XX disorder of sex development (DSD) associated with CAH. The patient carrying c.[293-13C>G];p.[(Ile173Asn)] pathogenic variants was a boy diagnosed at three years with CAH. At the time of the diagnosis, he presented with pubarche and increased growth rate; however, because of his gender and heterogeneous phenotype, pediatricians could not conclude whether he was an NC or SV form. The patient carrying p.[(Leu13Met;Ile173Asn)];[(Glu319*)] was a girl who presented with vomiting and dehydration after birth, together with clitoromegaly. She immediately started treatment with hydrocortisone and was surgically treated for clitoromegaly at eight years old. The genotype and phenotype information is presented in Table 2.

### 3.3. Genetic Studies of Relatives

Twenty-four blood relatives were submitted for genetic testing. Of them, 21 (87.5%) were carriers, 2 were wild type, and 1 sister was a compound heterozygote with mild symptoms of androgen excess and diagnosed with NC CAH. Interestingly, two daughters aged four and seven years old from a homozygous mother presented with the p.[(Val282Leu)];[(Gln319*)] genotype. Both alleles were inherited from their parents, and they were initially diagnosed with NC CAH despite presenting with normal 17-OHP and cortisol concentrations. However, after a 10-year follow-up, no symptoms were observed. The study of large rearrangements by MLPA eight years later allowed for the identification of a duplication of the paternal *CYP21A2* allele that compensated for the pathogenic genotype; therefore, they were reclassified as heterozygous carriers.

Ten male partners from couples with gestational desire were also submitted for genetic testing and counseling. Seven of them were wild type and three (30%) were heterozygous carriers of mild mutations. Considering that the CAH-affected females were all homozygous or compound heterozygous for NC forms, genetic counseling for pre-implantation studies was not considered.

### 3.4. Allelic Distribution of the Point Mutation Pathological Variants

In the 16 patients whose parents’ DNA was not available, the allelic distribution of the variants was studied by designed specific primers (Table 3). Of the 12 patients with two-point mutations, 11 presented their pathological variants in trans, confirming the clinical and biochemical diagnosis. The remaining patient presented the pathological variants in cis; therefore, the genetic diagnosis of CAH was ruled out. Indeed, this last patient was a boy clinically diagnosed with NC CAH at seven years of age. However, at the time of the genetic study, he was asymptomatic and free of medication. Of the four patients carrying three-point mutations, three of them carried two severe mutations in the same allele and one mild mutation p.(Val282Leu) in the other, giving rise to a NC phenotype. The remaining patient carried two severe mutations in both alleles, giving rise to a SW phenotype.

## 4. Discussion

The present study portrays the outcome of the molecular diagnosis of 59 patients with suspected CAH due to 21-OHD in a clinical laboratory setting over a 10-year period. The benefits of knowing the patient’s genotype include accurate phenotype prediction and appropriate clinical follow-up, identification of additional family members, and genetic and prenatal counseling if needed. Out of 59 referred patients, 37 were genetically positive for mutations in the *CYP21A2* gene. After clinical, biochemical, and genetic evaluations, 78.4% and 18.9% of patients presented with NC and CL forms, respectively, as in previously published results [21]. Previously published data regarding CL forms found a similar prevalence to the one found in our population [22,23,24,25]; however, because of the reduced number of CL patients in our cohort, conclusions regarding this group of patients should be cautiously interpreted.

Considering that CL forms manifest early in life, the mean age at diagnosis for the SV phenotype was unexpectedly high as a result of a patient born in 1940 and diagnosed at 33 years old with 46,XX DSD. No statistical differences were observed for NC forms versus patients with a negative genetic result regarding age at diagnosis. Interestingly, 93.1% of the NC forms corresponded to female subjects, reinforcing the idea that among the late-diagnosed patients, the majority are female. Despite the fact that the recognition of affected males has improved in recent years because of increased clinical awareness of CAH, our results lead to the idea that male patients with mild forms of CAH may still go underdiagnosed.

An overall phenotype-genotype correlation of 88.9% was observed, which resembles previously published data for other populations [26,27,28]. However, and in spite of this excellent genotype-phenotype correlation, four patients presented with discordancy, reinforcing the idea that the results of molecular testing alone should not be used to assign or change the CAH phenotype.

As in previously published results [28], 56.8% of our patients were compound heterozygotes, with disease severity being predicted by the less-severe mutation [29]. Among the 74 tested alleles, point mutations were found in 89.2% and large rearrangements were found in the rest. Overall, 19 different variants were found, with the mild p.(Val282Leu) variant being the most frequent one, followed by c.293-13C>G and large deletions or conversions. As described in other Mediterranean countries, 89.7% of our patients with a NC phenotype were either compound heterozygotes or homozygotes for the p.Val282Leu variant [9,21,30,31,32]. One new variant introducing tyrosine for histidine at position 283 of the protein was found in homozygosis. A positive genetic diagnosis in the patient was performed according to in silico programs. Clinically, the patient was diagnosed at 24 years old with menstrual ataxia and hirsutism, and was therefore classified as a NC form.

As expected, the clinical presentation was significantly different between the SW and SV phenotypes. Adrenal crisis was observed in only SW forms, whereas ambiguous genitalia were observed in both SW and SV forms. Regarding late-onset symptomatology, a clear overlap between NC forms and patients without a genetic diagnosis was observed. Several explanations can be raised on behalf of this fact regarding genetically negative patients. Firstly, some of these patients may carry pathogenic variants located in non-coding regions, such as introns or promoter areas not currently studied. In addition, there are other much rarer forms of CAH that could also be considered as causes of the symptomatology, including 11-β-hydroxylase deficiency, 17-α-hydroxylase deficiency, 3-β -hydroxysteroid dehydrogenase deficiency, congenital lipoid adrenal hyperplasia, and p450 oxidoreductase deficiency [33]; thus, for the remainder of the present work, CAH only indicates CAH due to 21-hydroxylase deficiency. The overlap may also occur because heterozygote carriers (half of the patients with negative genetic diagnoses) may present with clinical and biochemical features like those observed with the NC phenotype, as previously suggested [34]. Finally, the clinical features of NC forms and ovarian disturbances, such as polycystic ovarian syndrome (POCS), are similar [35]. Considering that 91% of the non-genetically diagnosed patients were females, a diagnosis of PCOS should also be contemplated. In that sense, a previous work from our group established that more than a third of the women followed with NC CAH could not be genetically diagnosed, suggesting that misclassification of NC forms is common [13].

Biochemically, 17-OHP concentrations were significantly higher in genetically confirmed patients; however, no statistical analysis was performed among CAH groups regarding 17-OHP concentration because of a reduced number of patients with CL forms and a fixed upper limit of quantification at 60.6 nmol/L. It would have been of interest to see if those variants causing <2% of enzymatic activity presented with higher 17-OHP concentrations. Nonetheless, because of the clinical nature of the current study and the clinic irrelevance of the 17-OHP concentrations above 60.6 nmol/L, the serum samples were not diluted. After stimulation with synthetic ACTH, 17-OHP concentrations were statistically higher for patients with a NC form when compared to genetically negative patients. Interestingly, and as previously reported (Bidet 2009), patients carrying a mutated allele presented a tendency toward higher concentrations of 17-OHP. However, 17-OHP concentrations were in all cases under 30 nmol/L, reinforcing the idea that in those patients with 17-OHP concentrations below 30 nmol/L after stimulation with cosyntropin, we can avoid practicing the genetic study.

The inclusion of relatives in the study revealed one further case of NC CAH. Interestingly, 87.5% of relatives were carriers of a pathogenic variant, including two siblings initially classified as genetically positive. One of the pathogenic variants carried by the girls was p.(Gln319*), which has been found to be associated with functional duplications of the *CYP21A2* gene in around 84% of patients [36]. Hence, the introduction of MLPA analysis in the genetic approach of 21-OHD is vital if a misdiagnosis is to be avoided. In addition, the study of male partners with gestational desire identified several carriers of mild mutations. However, because of the mild nature of the pathogenic variants, neither pre-implantational nor prenatal diagnosis were considered. Overall, and considering that a higher frequency of CAH has been described in the Mediterranean region [2,37], as well as the high frequency of carriers in the Spanish population [7], studying partners from affected patients with gestational desire seems advisable.

Finally, the study of the allelic distribution of the variants allowed for reclassifying one patient from affected to carrier. This is critical for the molecular diagnosis of 21-OHD, as a possible misdiagnosis could come from the erroneous identification of a wild-type allele as pathological. Studying the allelic distribution also allowed for phenotype prediction in four patients carrying three mutations.

The present study resulted from an unprogrammed clinical practice setting and therefore several limitations exist, mainly because of the retrospective manner in which it was conducted. One is the heterogeneous degree of clinical information available according to that supplied by the clinician. In addition, the biochemical data are in some cases incomplete. Regarding technical pitfalls, the measurement of 17-OHP by immunoanalysis procedures may have interferences, and the over-measurement of its values may occur, especially in NC forms. Genetically, and despite no effect on the phenotype prediction, the use of MLPA does not discriminate between gene deletions or conversions to the pseudogene [38]. Some authors also suggest that the clinical presentation of CAH should only be classified as CL or NC. According to their criteria, the subdivision of CL CAH into SW and SV forms should be avoided, because all patients lose salt to some degree [39]. Finally, our cohort has a limited number of patients compared to similar works in other populations, especially regarding CL forms.

## 5. Conclusions

In conclusion, our report expands the existing molecular information relating to CAH patients due to *CYP21A2* mutations in the Spanish population. Considering the high genetic heterogeneity of the RCCX module, a genetic approach including Sanger sequencing, MLPA analysis, and allelic distribution of the pathogenic variants represents a beneficial tool for better classification of patients with 21-OHD.

## Figures and Tables

**Table 1:** Summary of clinical, biochemical, and genetic information of patients at diagnosis.

	Classic SW	Classic SV	NC	Non-Definite	Negative Genetic Diagnosis
Patients *n* (%)	5 (8.5)	2 (3.4)	29 (49.2)	1 (1.7)	22 (37.9)
Age at diagnosis in years, mean (SD)	0.043 (0.035)	16.5 (21.9)	21.3 (10.9)	3	15.7 (9.1)
Female (%)	3 (60)	2 (100)	27 (93.1)	0 (0)	20 (91)
Clinical features at presentation, *n* (%)					
Adrenal crisis	5 (100.0)	0	-	-	-
Atypical genitalia	3 (60.0)	2 (100.0)	-	-	2 (9.1)
Early puberty	-	-	6 (20.7)	1 (100)	3 (13.6)
Hirsutism	-	-	7 (24.1)	-	4 (18.2)
Acne	-	-	1 (3.4)	-	1 (4.5)
Menstrual abnormalities	-	-	3 (10.3)	-	4 (18.2)
Infertility	-	-	7 (24.1)	-	2 (9.1)
17-OHP concentration (nmol/L)					
17-OHP (basal), median (IQR)	60.6 (60.6–60.6)	60.6 (60.6–60.6)	41.2 (19.8–59.2)	60.6	6.2 (3.1–8.8)
17-OHP (60 min post ACTH), median (IQR)	-	-	60.6 (60.6–60.6)	-	16.3 (7.7–19.8)
Mutation status *n* (%)					
Homozygosis	-	-	10 (34.5)	-	-
Compound Heterozygosis	2 (40.0)	2 (100.0)	16 (55.2)	1 (100.0)	-
Hemizygosis	3 (60.0)	-	3 (10.3)	-	-
Heterozygote carriers	-	-	-	-	11 (50.0)
Wild type	-	-	-	-	11 (50.0)

SW—salt-wasting; SV—simple-virilizing; NC—non-classical.

**Table 2:** Genotype and expected and observed phenotypes in genetically confirmed patients.

Group	Enzyme Activity	Pathogenic Variants (DNA)	Pathogenic Variants (Protein)	Proband	Number of Patients	Expected Phenotype	Observed Phenotype
0	Null	c.[(-1)_(202+1_203-1)del];[(-1)_(202+1_203-1)del;(651+1_652-1)_(313+1_314-1)del]	p.[?];[?]	Compound Hz	1	SW	SV
A	<2%	c.[293-13C>G];[(-1)-(1118+1_119-1)del]	p.[?];[?]	Hemizygote	2	SW	SW
		c.[1069C>T];[(-1)-(1118+1_119-1)del]	p.[(Arg357Trp)];[?]	Hemizygote	1	SW	SW
		c.[293-13C>G];[874G>A]	p.[?];[(Gly292Ser)]	Compound Hz	1	SW	SW
		c.[293-13C>G];[710T>A;713T>A;719T>A]	p.[?];[(Ile237Asn;Val238Glu;Met240Lys)]	Compound Hz	1	SW	SV
B	~2%	c.[293-13C>G];[518T>A]	p.[?];[(Ile173Asn)]	Compound Hz	1	SV	NC/SV
		c.[37C>A;518T>A];[955C>T]	p.[(Leu13Met;p.Ile173Asn];[(Gln319*)]	Compound Hz	1	SV	SW
C	~20–60%	c.[844G>T];[844G>T]	p.[(Val282Leu)];[(Val282Leu)]	Hm	9	NC	NC
		c.[844G>T];[(-1)-(1118+1_119-1)del]	p.[(Val282Leu)];[?]	Hemizygote	3	NC	NC
		c.[844G>T];[955C>T]	p.[(Val282Leu)];[(Gln319*)]	Compound Hz	1	NC	NC
		c.[844G>T];[1280G>A]	p.[(Val282Leu)];[(Arg427His)]	Compound Hz	1	NC	NC
		c.[844G>T];[1360C>T]	p.[(Val282Leu)];[(Pro454Ser)]	Compound Hz	1	NC	NC
		c.[844G>T];[1451_1452delinsC]	p.[(Val282Leu)];[(Arg484Profs*58)]	Compound Hz	1	NC	NC
		c.[844G>T];[ 955C>T; 1451_1452delinsC]	p.[(Val282Leu;Gln319*];[(Arg484Profs*58)]	Compound Hz	1	NC	NC
		c.[92C>T];[844G>T]	p.[(Pro31Leu)];[(Val282Leu)]	Compound Hz	2	NC	NC
		c.[92C>T];[518T>A]	p.[(Pro31Leu)];[(Ile173Asn)]	Compound Hz	1	NC	NC
		c.[332_339del];[844G>T]	p.[(Gly111Valfs*21)];[(Val282Leu)]	Compound Hz	1	NC	NC
		c.[293-13C>G];[844G>T]	p.[?];[(Val282Leu)]	Compound Hz	1	NC	NC
		c.[293-13C>G];[844G>T]	p.[?];[(Val282Leu)]	Compound Hz	3	NC	NC
		c.[293-13C>G;332-339del];[844G>T]	p.[(?; Gly111Valfs*21)];[(Val282Leu)]	Compound Hz	1	NC	NC
		c.[293-13C>G;1360C>T];[844G>T]	p.[(?; Pro454Ser)];[(Val282Leu)]	Compound Hz	1	NC	NC
		c.[844G>T];[*13G>A]	p.[?];[(Val282Leu)]	Compound Hz	1	NC	NC
D	Unknown	c.[847C>T];[847C>T]	p.[(His283Tyr)];[(His283Tyr)]	Hm	1	-	NC

Hm—homozygote; Hz—heterozygote; SW—salt-wasting; SV—simple-virilizing; NC—non-classical.

**Table 3:** Allelic distribution of the point-pathological variants in patients without their parents’ genetic data.

Patient	Cis/Trans	Genotype	Phenotype
1	trans	c.[293-13C>G];p.[(Ile237Asn;Val238Glu;Met240Lys)]	SV
2	trans	c.[293-13C>G];p.[(Val282Leu)]	NC
3	trans	c.[293-13C>G];p.[(Val282Leu)]	NC
4	trans	c.[293-13C>G];p.[(Val282Leu)]	NC
5	trans	c.[293-13C>G];p.[(Ile173Asn)]	NC
6	cis	[c.293-13C>G;p.(Pro454Ser)]	NC
7	trans	p.[(Val282Leu)];[(Pro454Ser)]	NC
8	trans	p.[(Pro31Leu)];[(Val282Leu)]	NC
9	trans	p.[(Pro31Leu)];[(Val282Leu)]	NC
10	trans	p.[(Val282Leu)];[(Arg427His)]	NC
11	trans	p.[(Gly111Valfs*21)];[(Val282Leu)]	NC
12	trans	c.[13*G>A];p.[(Val282Leu)]	NC
13	trans	[c.293-13C>G;p.(Gly111Valfs*21)];[p.(Val282Leu)]	NC
14	trans	[c.293-13C>G;p.(Pro454Ser)];[p.(Val282Leu)]	NC
15	trans	p.[(Val282Leu)];[(Gln319*;Arg484Profs*58)]	NC
16	trans	p.[(Leu13Met;Ile173Asn)];[(Glu319*)]	SW

## Data Availability

Data is contained within the article or supplementary material.

## Supplementary Materials

The following are available online at https://www.mdpi.com/2077-0383/10/6/1183/s1, Table S1: Designed primer sequences for the CYP21A2 analysis; Table S2: Treatment and clinical manifestations during the follow-up of patients diagnosed with HSC due to *CYP21A2* mutations, Figure S1: Primer sequence containing one of the variants detected in the patient.
