# Supplementary material for: Comprehensive Genetic Testing of CYP21A2: A Retrospective Analysis in Patients with Suspected Congenital Adrenal Hyperplasia
Source: J Clin Med. 2021 Mar 12;10(6):1183. doi: 10.3390/jcm10061183 (PMC8001222; doi:10.3390/jcm10061183)
Supplement: Supplementary file 1 [file jcm-10-01183-s001.pdf]

Table S1. Designed primer sequences for the CYP21A2 analysis.

Fragment	SNP that Differ from the Gene and the Pseudogene	Primer Sequence	Complementary Primer Sequence
1	-296T>C, -295A>C, -284A>G, -282T>G	5′-GACACTATTGCCTGCACAGT-3′	5′-GTAGTCTCCCAAGG-3′
1	c.332_339delGAGACTAC	5′-GTAGTCTCCCAAGG-3′	5′-GACACTATTGCCTGCACAGT-3′
2	c.293-104delG	5′-GGGCATATCTGGTGGGGAGA-3′	5′-AAGGAGAAACTGAGGTA-3′

Figure S1. Primer sequence containing one of the variants detected in the patient.

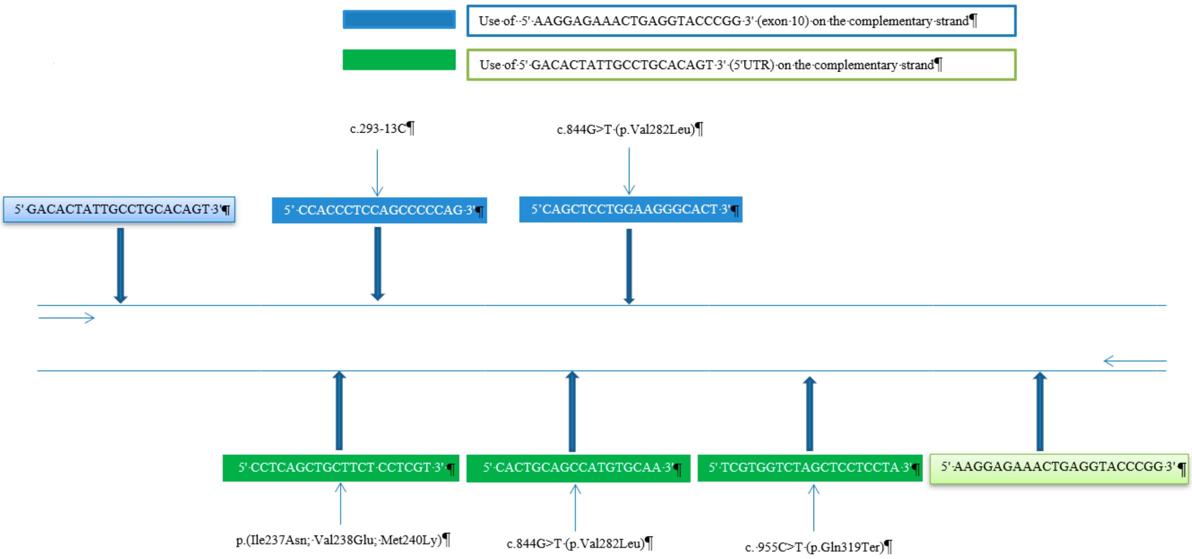

Mutation included in the primer sequence	Primer sequence	Complementary primer sequence
c.[710T>A;713T>A;719T>A] (p.Ile237Asn; Val238Glu; Met240Lys)	5' CCTCAGCTGCTTCTCCTCGT 3' (exon 6)	5' GACACTATTGCCTGCACAGT 3' (5'UTR)
c.293-13C	5' CCACCCTCCAGCCCCCAG 3' (intron 2)	5' AAGGAGAAACTGAGGTACCCGG 3' (exon 10)
c.844G>T (p.Val282Leu)	5' CACTGCAGCCATGTGCAA 3' (exon 7)	5' GACACTATTGCCTGCACAGT 3' (5'UTR)
c.844G>T (p.Val282Leu)	5' CAGCTCCTGGAAGGGCACT 3' (exon 7)	5' AAGGAGAAACTGAGGTACCCGG 3' (exon 10)
c. 955C>T (p.Gln319Ter)	5' TCGTGGTCTAGCTCCTCCTA 3' (exon 8)	5' GACACTATTGCCTGCACAGT 3' (5'UTR)

Table S2. Treatment and clinical manifestations during the follow-up of patients diagnosed with HSC due to *CYP21A2* mutations.

Form	Classical	Non-Classical	Non-Definite
Follow-up	5 Yes<br>2 No	16 Yes<br>12 No (one patient in treatment with dexamethasone, the rest with ACOS or without treatment)<br>1 Recently diagnosed	Yes
Treatment at the time of diagnosis	5 Hydrocortisone, Fludrocortisone, NaCl (SW)<br>1 Dexamethasone (SV)<br>3 Genitoplasty	14 Dexamethasone<br>4 Oral contraceptives<br>2 Prednisone<br>7 No treatment	Dexamethasone, Fludrocortisone
Treatment during follow-up	4 Hydrocortisone, Fludrocortisone<br>2 Hydrocortisone<br>1 Dexamethasone (instead of hydrocortisone)<br>1 Dexamethasone, Fludrocortisone (instead of Hydrocortisone, Fludrocortisone)<br>1 Dexamethasone, required only for stressful situations	7 Dexamethasone<br>3 Prednisone<br>4 Oral contraceptives<br>1 Hydrocortisone<br>2 Hydrocortisone, required only for stressful situations<br>11 No treatment	Dexamethasone
Clinical manifestations during follow-up	2 Hirsutism<br>3 Menstrual abnormalities<br>1 Hyperandrogenism<br>4 Atypical genitalia<br>1 46,XX Disorder of sex development<br>1 Infertility<br>2 No relevant symptoms yet (two boys of 1 and 9 years old)	10 Hirsutism<br>3 Menstrual abnormalities<br>4 Infertility<br>1 Accelerated bone age<br>2 Acne<br>1 Early puberty<br>9 Asymptomatic	Analytical alterations (elevated 17-OHP and low renin concentrations)
